# Can we see the world the same? Interdisciplinarity in palliative care

**DOI:** 10.15649/cuidarte.2568

**Published:** 2022-09-01

**Authors:** Liana Magreth Peñaranda Ospina, Fabio Stefant Iglesias Meza, Alejandra Maria Alvarado Garcia

**Affiliations:** 1 Universidad Cooperativa de Colombia, Bucaramanga, Colombia. Email: liana.penaranda@campusucc.edu.co correspondence author Universidad Cooperativa de Colombia Universidad Cooperativa de Colombia Bucaramanga Colombia liana.penaranda@campusucc.edu.co; 2 Hospital Geriátrico San Miguel Cali, Colombia. Email: figlesias02@uan.edu.co Pontificia Universidad Javeriana Hospital Geriátrico San Miguel Cali Colombia figlesias02@uan.edu.co; 3 Universidad Antonio Nariño, Bogotá, Colombia. Email: alalvarado39@uan.edu.co Universidad Antonio Nariño Universidad Antonio Nariño Bogotá Colombia alalvarado39@uan.edu.co

According to the World Health Organization (WHO), palliative care strives to improve the quality of life of persons and their families who face physical, psychological, social, or spiritual challenges associated with life-threatening illness^1^. Worldwide, it is estimated that about 40 million people need palliative care, but only 14% receive it^2^. The WHO also indicates that 78% of people who need palliative care live in developing countries^2^, which reveals a great need for palliative care not only for terminally-ill people but also for their families.

The need for palliative care worldwide goes hand in hand with the increase in aging, which progressively and almost simultaneously converges with manifestations of organic exhaustion and processes of health and disease framed in the individual’s environment and biography. However, it should not be overlooked that people of any age and at any stage of serious illness are candidates for palliative care and may require different approaches to care depending on the individuals’ needs^3^. Genetics-based knowledge has allowed the hypothesis of life, but the individual’s environment is influenced from before birth. Nutritional history, tobacco use, exposure to environmental toxicants, and life cycle have been defined as causes of epigenetic alterations, which may contribute to diseases amenable to palliative care in advanced stages.

These changes -which in general mark a society’s development measured by increased life expectancy− have a population group that is growing silently within them. Some people are born with severe impairments that make life difficult for themselves and their families, others whose lives are unexpectedly cut short, and another group that grows significantly but more predictably: the aging population. For these people, the WHO proposes several objectives aimed at improving the quality of life and providing comfort, taking into account even more symptom severity and variability, being essential that health personnel assess the needs of these people and their families in the physical, emotional, social, spiritual, and health support areas^4^.

This is why interdisciplinarity in palliative care is needed for people with advanced and incurable diseases, understanding interdisciplinarity as the exchange of experiences and competencies between groups of different health professionals^5^. Two sets of knowledge (doctors’ and nurses’) are no longer sufficient, and no therapeutic approach has been developed to control pain, unpleasant symptoms, and suffering of people and their families. Therefore, it is necessary to integrate different professionals to provide competent palliative care focused on symptoms management, allowing uniform care considered from different perspectives, promoting comprehensive and dignified care, and ensuring necessary conditions to provide palliative and end-of-life care^6^.

Studies in the literature show that interdisciplinary palliative care allows people to process this new phase of care, including the use of time and space mediated by a comprehensive clinical team that extends palliative care to family^7^ and caregivers, reduces caregivers’ burden, and improves coordination of care. Likewise, interdisciplinary palliative care enhances the well-being of individuals as all members of the interdisciplinary team are moving in the same direction, and even they are instrumental in identifying the needs of people who may require early palliative care^8^.

Each professional intervenes from their knowledge, and when they are brought together, the perspective of care and the possibility of creating comprehensive care models focused on the specific requirements of patients in palliative care and their families change. Therefore, it is essential to build positive interpersonal relationships in which each member is listened to, contributing to the identification of needs and the development of individualized strategies provided at the right time and for the necessary time. The idea is not only to integrate traditional medicine but also to offer strategies from an interdisciplinary approach that complement the action^9^, contributing significantly to the control of unpleasant signs and symptoms and optimizing comfort measures.

Achieving competent and interdisciplinary palliative care involves recognizing the importance of each discipline and its contribution not only to symptom management but also to the development of care plans and decision making. Each professional should be clear about their role within the team. They should be able to participate actively, listen and be listened to, have respect for other disciplines as a guiding principle, and internalize the objectives of palliative care. Furthermore, they should put aside professional ego, avoid confrontations, and achieve an assertive communication in which the knowledge and experience of each member are inclusively evidenced and integrate with the patient-family dyad care^10^.

Thinkingofpalliativecareguidedbyasingledisciplinewouldbetoignorethecomprehensiveness and individuality of the needs for control of signs and symptoms of people in a situation characterized by producing multiple symptoms. It is essential to propose models that involve all disciplines, those in the field of health (medicine, nursing, rehabilitation, psychology, social work, gerontology, dentistry, nutrition) and those in specific areas that study the environment that affects human being well-being (ethnography, sociology, anthropology, history, economics, among others). In this way, it is possible to thoroughly understand the health-disease process, integrating the physical, social and cultural spheres in a given context^11^.

There will always be the need to continue building on the health-disease process without neglecting its social connotation, individual trajectories, cultural symbols, social context, and historical determination. The health-disease process, therefore, calls for the participation of different disciplines that can be integrated from their specific vision to help people receiving palliative care and their families to obtain a level of comfort and -individual and family− well- being and thus reduce suffering during the end-of-life stages^12^.
